# Commercial genetic testing for type 2 polysaccharide storage myopathy and myofibrillar myopathy does not correspond to a histopathological diagnosis

**DOI:** 10.1111/evj.13345

**Published:** 2020-10-29

**Authors:** Stephanie J. Valberg, Carrie J. Finno, Marisa L. Henry, Melissa Schott, Deborah Velez-Irizarry, Sichong Peng, Erica C. McKenzie, Jessica L. Petersen

**Affiliations:** 1 Large Animal Clinical Sciences, College of Veterinary Medicine, Michigan State University, East Lansing, MI, USA; 2 Department of Population Health and Reproduction, School of Veterinary Medicine, University of California-Davis, Davis, CA, USA; 3 Department of Clinical Sciences, College of Veterinary Medicine, Oregon State University, Corvallis, OR, USA; 4 Department of Animal Science, University of Nebraska-Lincoln, Lincoln, NE, USA

**Keywords:** horse, muscle disease, glycogen, skeletal muscle, validation

## Abstract

**Background:**

Commercial genetic tests for type 2 polysaccharide storage myopathy (PSSM2) and myofibrillar myopathy (MFM) have not been validated by peer-review, and formal regulation of veterinary genetic testing is lacking.

**Objectives:**

To compare genotype and allele frequencies of commercial test variants (P variants) in *MYOT* (P2; rs1138656462), *FLNC* (P3a; rs1139799323), *FLNC* (P3b; rs1142918816) and *MYOZ3* (P4; rs1142544043) between Warmblood (WB) and Arabian (AR) horses diagnosed with PSSM2/MFM by muscle histopathology, and phenotyped breed-matched controls. To quantify variant frequency in public repositories of ancient and modern horse breeds.

**Study design:**

Cross sectional using archived clinical material and publicly available data.

**Methods:**

We studied 54 control-WB, 68 PSSM2/MFM-WB, 30 control-AR, 30 PSSM2/MFM-AR and 205 public genotypes. Variants were genotyped by pyrosequencing archived DNA. Genotype and allele frequency, and number of variant alleles or loci were compared within breed between controls, PSSM2/MFM combined and MFM or PSSM2 horses considered separately using additive/genotypic and dominant models (Fisher’s exact tests). Variant frequencies in modern, early domestic and Przewalski horses were determined from a public data repository.

**Results:**

There was no significant association between any P locus and a histopathological diagnosis of PSSM2/MFM, and no difference between control and myopathic horses in total loci with alternative alleles, or total alternate alleles when PSSM2/MFM was considered combined or separately as PSSM2 or MFM. For all tests, sensitivity was <0.33. Allele frequencies in WB (controls/cases) were: 8%/15% (P2), 5%/6% (P3a/b) and 9%/13% (P4); in AR, frequencies were: 12%/17% (P2), 2%/2% (P3a/b) and 7%/12% (P4). All P variants were present in early domestic (400- to 5500-year-old) horses and P2 present in the Przewalski.

**Conclusions:**

Because of the lack of significant association between a histopathological diagnosis of PSSM2 or MFM and the commercial genetic test variants P2, P3 and P4 in WB and AR, we cannot recommend the use of these variant genotypes for selection and breeding, prepurchase examination or diagnosis of a myopathy.

## 1 | INTRODUCTION

Equine polysaccharide storage myopathy (PSSM) is one specific form of exertional rhabdomyolysis (ER) that was originally identified in Quarter Horses and related breeds. Hallmarks used to discover PSSM were aggregates of amylase-resistant polysaccharide in periodic acid-Schiff’s (PAS) stains of skeletal muscle and muscle glycogen concentrations greater than 1.5-fold that of normal muscle.^1^ The diagnostic criteria for PSSM subsequently broadened to include amylase-sensitive aggregates of polysaccharide; this expanded definition resulted in a wider diversity of breeds diagnosed with PSSM.^2^ In 2008, a dominant, nonsynonymous gain-of-function mutation in the glycogen synthase 1 (*GYS1*) gene was identified, refining the classification of PSSM. Horses with PSSM attributed to the presence of the *GYS1* variant have since been classified as having type 1 PSSM (PSSM1).^3^ The term type 2 PSSM (PSSM2) therefore was developed as a descriptor for any horse that has clinical signs of exercise intolerance and abnormal aggregates of polysaccharide in muscle fibres that does not possess the *GYS1* mutation.^4,5^ PSSM2 is the most common form of PSSM in Warmblood (WB) and Arabian (AR) horses at >80% and 100% of PSSM cases, respectively, whereas PSSM1 predominates in the continental European-derived draught breeds, Quarter Horses and related stock breeds.^6,7^

Recently, a more in-depth histological analysis of muscle biopsies from AR and WB with PSSM2 revealed that a subset of those horses had abnormal aggregates of the cytoskeletal protein desmin in a small fraction of mature type 2A muscle fibres.^8,9^ In electron micrographs from these horses, glycogen was found to pool between disarrayed myofibrils.^8,9^ Based on these findings, the subset of AR and WB with desmin aggregates that had originally been diagnosed with PSSM2 was reclassified as having a myofibrillar myopathy (MFM). In WB and AR, the similarity in clinical signs in both MFM and PSSM2 supports PSSM2 being an earlier manifestation of MFM^8–10^; however, it has not yet been firmly established. MFM is a relatively rare myopathy in humans.^11–13^ In humans, mutations in genes that encode proteins in the Z-disc of the sarcomere [*DES, PLEC 1, CRYAB, FLNC, MYOT, ZASP, BAG3, FHL1 and DNAJB6*] are well-described causes of MFM, with eight other genes causing MFM-like disorders.^14^ In 50% of human MFM cases, however, the molecular basis remains unknown.^13,14^

In horses and other domestic species, genetic tests are commercially available for use in diagnosing and managing disease and to inform selection and breeding decisions. For myopathies, such as hyperkalaemic periodic paralysis (HYPP), PSSM1, glycogen branching enzyme deficiency (GBED), malignant hyperthermia (MH) and myosin heavy chain myopathies (MYHM), the frequency of each associated mutation is well described in diseased and healthy populations, and functional data regarding the effect of each mutation are reported in peer-reviewed publications.^3,15–19^ In human medicine, the American College of Medical Genetics and Genomics (ACMGG) recommends association or segregation analyses in well-phenotyped individuals, as well as modelling and functional studies as necessary means to determine that variants identified through sequencing are actually causative of disease.^20^ Although there are similar principles for the use of genetic information in commercial venues agreed upon by the equine genetic research community, unlike in human medicine, there is no regulation of veterinary genetic testing [consensus statement on the translation and application of genomics in equine industries: Horse Genome Project, https://horsegenomeworkshop.com/values]. Analyses that determine the frequency of a purported causative variant in populations of both healthy horses and those diagnosed with the disease using the gold standard for diagnosis provide vital information about the accuracy of using the mutation as a diagnostic or management tool.

Commercial genetic testing has recently been offered for PSSM2 and MFM in horses using single-nucleotide polymorphism (SNP) variants in genes myotilin (*MYOT*), filamin C (*FLNC*) and another Z-disc protein not previously reported to cause MFM, myozenin (*MYOZ3*) (Equiseq.com, centerforanimal genetics.com). The variant (nonreference) alleles in the commercial test are termed ‘P2’ for that in *MYOT*, ‘P3a’ and ‘P3b’ for those in *FLNC* and the variant in *MYOZ3* is annotated as ‘P4’. The inference from the test developers is that cases of PSSM2 are an early stage of MFM (http://equiseq.com/learning_center/health/myofibrillar-myopathy-mfm). Unlike HYPP, PSSM1, GBED, MH and MYHM, there are no peer-reviewed publications available to validate the scientific basis and diagnostic utility of the developers’ PSSM2/MFM genetic tests. Our first objective was to compare genotype and allele frequencies of the four variants used in commercial genetic testing in WB and AR horses diagnosed with MFM or PSSM2 by muscle histopathology compared to those of breed-matched, healthy controls. Additionally, we sought to determine allele frequencies at each of the P loci in publicly available data including a variety of early equids and modern horse breeds.

## 2 | MATERIALS AND METHODS

### 2.1 | Case selection

The database of the Neuromuscular Diagnostic Laboratory, Michigan State University was searched to identify WB and AR horses with a previous diagnosis of MFM, PSSM2 or both based on muscle histopathology. Horses that were diagnosed with PSSM2 or MFM or both were then classified as PSSM2/MFM. All horses identified with MFM were selected for inclusion. Horses with a diagnosis of PSSM2 and no evidence of MFM were selected for inclusion if they had DNA already isolated. A diagnosis of MFM was based on the presence of desmin-positive cytoplasmic aggregates in four or more mature myofibres (~<1% of fibres/biopsy). Regenerative fibres with dark desmin staining rather than aggregates of desmin were excluded from diagnostic criteria for MFM. Regenerative fibres were identified by large internalised myonuclei and basophilic cytoplasm in haematoxylin and eosin stains. This is a less stringent criterion than that of six or more fibres with desmin aggregates used in a previous MFM study^8^ and was adopted to include all potential cases of MFM. Desmin staining had been previously performed on all PSSM2/MFM-AR cases and was performed on any PSSM2- or control-WB that had not previously been analysed in this manner. PSSM2 was diagnosed based on the presence of PAS-positive cytoplasmic inclusions in muscle fibres and in WB, a negative result for the *GYS1* mutation. *GYS1* testing had previously been performed and was negative for 12 PSSM2/MFM-AR and not pursued further because the *GYS1* mutation is not reported in this breed.^21^

### 2.2 | Control selection

The Neuromuscular Diagnostic Laboratory, Michigan State University database was screened to identify control horses ≥2 years of age from previous research studies that were negative for the *GYS1* mutation and had normal gluteal or semimembranosus muscle histopathology on frozen sections stained with a minimum of haematoxylin and eosin, desmin, PAS and amylase-PAS stains. Samples that were previously obtained from healthy AR horses housed on several farms across the United States were used as controls (n = 30). The repository contained muscle biopsies from 10 healthy WB from a variety of farms for inclusion as control-WB. To supplement the number of control-WB, additional WB were selected from samples submitted by referring veterinarians to the Neuromuscular Diagnostic Laboratory, from across the United States with the criteria that there were no evident histopathological findings in haematoxylin and eosin, modified Gomori Trichrome, nicotinamide adenine dinucleotide tetrazolium reductase, oil red O, PAS, amylase-PAS and desmin stains. Preference was given to samples from horses with a history consistent with shivers or stringhalt because these are not muscle diseases.^22,23^ There were 11 control-WB that had no clinical signs of muscle disease, 16 with a primary complaint of shivers/stringhalt, 10 with a hindlimb gait abnormality (ataxia, weakness and lameness), seven with atrophy/fasciculations, five with poor performance, three with ER and two with muscle soreness.

### 2.3 | DNA isolation and polymerase chain reaction

DNA was isolated from hair bulbs, buffy coat, whole blood or muscle tissue using Gentra Puregene Kits, and DNA quality/quantity evaluated using a Picogreen assay. Thermocycling was carried out using recombinant AmpliTaq^s^ Gold polymerase in a PTC-200 thermocycler, and the PCR products run on a 1.0% agarose gel for visualisation.

### 2.4 | Pyrosequencing

The patent application at https://patents.google.com/patent/WO2017165733A1/en was used to identify the genomic coordinates of the variants as listed in EquCab2.0. The impact of each mutation was predicted using Sorting Intolerant From Tolerant analysis (SIFT).^24^ Primers (Table S1) were designed using the PyroMark Assay Design Software 2.5.8 and EquCab2.0 reference genome, based on the patent application coordinates, with the sequence and coordinates confirmed in EquCab3 (Table 1). The reverse primer was biotinylated using Biotin-TEG and HPLC purification (IDT, Coralville, Iowa, USA). The resulting biotin-labelled amplicons were annealed with the sequencing primer and the relative expression of each allele was determined by pyrosequencing as described in Kreutz et al.^25^ Briefly, PCR products were diluted in a solution containing 4 ul of streptavidin-coated sepharose beads and 40 ul of binding buffer (10 mM Tris-HCL, 2 M NaCl, 1 mM EDTA and 0.1% Tween^™^ 20 pH 7.6). Immobilised biotin-labelled amplicons were captured using a vacuum prep tool, washed and denatured to remove unbound primers and unbiotinylated strands using three solutions (ie 70% ethanol, denaturing solution containing 0.2 M NaOH and wash buffer containing 10 mM Tris-Acetate pH 7.6). Only the template strands remained bound after the washing steps. Sepharose beads with bound strands were diluted in annealing buffer (20 mM Tris-Acetate and 5 mM MgAc_2_ pH 7.6) containing the sequencing primer and pyrosequenced (PyroMark Gold Q96 reagents and PSQ 96MA pyrosequencer, Biotage, Charlotte, North Carolina, USA). Relative levels of each allele were quantified with the PyroMark AQ 2.5.8 software. All sample plates were analysed with positive controls consisting of horses with the alternate allele identified from RNA-seq data (GEO Series accession number GSE10 4388) and a template control containing no DNA. Resulting data were visually evaluated to determine genotype and allele frequencies. Genotypes were verified across all four variants in three horses by Sanger sequencing.

### 2.5 | Publicly available databases

The frequency of the reference and alternate alleles for the P2, P3a, P3b and P4 loci was determined in all horses identified by searching the NCBI Sequence Read Archive (https://www.ncbi.nlm.nih.gov/sra) using the query: “Equus caballus”[Organism] OR equus caballus[All Fields]) AND “biomol dna”[Properties] AND “strategy wgs”[Properties] AND “platform illumina”[Properties] AND sra_nuccore_alignment [Filter]. For instances where the genome build differed in reference or query databases, the appropriate coordinates were obtained using NCBI Coordinate Remapping Service (https://www.ncbi.nlm.nih.gov/genome/tools/remap/). A minimum coverage of five reads was required at each locus to call genotypes, which was performed using SRA tools pileup stats. Samples without sufficient coverage at a given locus were excluded from allele frequency calculations. The horses represented 25 breeds as well as ‘early domestic’ (400–5500 years old) samples, one ancient (24 000 years old) horse and Przewalski horses (*Equus ferus przewalskii*) (Table S2). Allele frequencies for early domestic, ancient and Przewalski horses were calculated together and by age of sample.

### 2.6 | Data analysis

Data analysis was performed at two separate Universities (University of California, Davis and University of Nebraska, Lincoln) and reviewed by an author at a third University (Oregon State University) independent from the author who generated the histopathological diagnosis (S.J.V., Neuromuscular Diagnostic Laboratory). A post-hoc statistical power analysis was performed to determine if our sample sizes were adequate to detect a significant effect, using a medium (d = 0.5) effect size. For the WB population, power was calculated as 99.6%. For the AR population, post-hoc power was calculated at 86.6%. Age data that were non-normally distributed by Shapiro-Wilk testing were log_10_ transformed and compared between respective control and myopathic groups using an unpaired t-test.

Initially, genotypes at each locus (homozygous reference vs. heterozygous, homozygous alternate) were compared using a Fisher’s exact test between two subgroups of control WB, both of which had no evident muscle histopathology. The first subgroup consisted of control horses with no potential overlapping clinical signs of PSSM2/MFM. This included horses with no clinical signs (N = 11) combined with control horses with signs of shivers/stringhalt (N = 16; total N = 27). Comparison was made to a second subset of the control group consisting of WB with clinical signs that overlap those of PSSM2/MFM (N = 27) [weakness, lameness, atrophy/fasciculations, poor performance, muscle soreness and ER]. Finding no significant difference in genotypes between the two subsets of control WB, further analyses were performed using the entire control WB group that had no evident muscle histopathology.

Control WB and control-AR horses were compared to a combined group of PSSM2 and MFM horses (PSSM2/MFM) by breed (Table 2). Control horses were also compared within breed to: 1) a PSSM2 group, which included all PSSM2 horses with or without MFM and 2) an MFM group, which included all MFM horses with or without PSSM2. Genotype and allele frequencies were calculated and compared between control and myopathic groups for each of the four variants within each breed using a Fisher’s exact test; additive and dominant models were considered. Out of concern that some WB control horses were younger than the potential age of onset for MFM, we also compared PSSM2/MFM-WB genotypes (homozygous reference to heterozygous, homozygous variant) at each locus between a subset of the control WB horses < 7 years of age (N = 20) to a subset ≥ 7 years of age (N = 34) using a Fisher’s exact test. For an allelic Fisher’s exact test, at least five individuals (ie 10 alleles) are required in both disease and control groups to identify a significant association (*P* < .05) assuming an autosomal dominant mode of inheritance.

The term variant was used to define an alternate (nonreference) allele at any P locus. The total number of P variant alleles per horse across all loci was counted considering P3 as a single locus, as P3a and P3b were in complete linkage disequilibrium in these samples. Total variant allele count per horse was classified then as none, one or >1 (maximum of 6 if the horse was homozygous across P2, P3 and P4) for contingency table analyses (Fisher’s exact test) to compare control vs. myopathic groups within each breed. Counts of total loci with a P variant (0 to 3, where 3 = at least one variant at P2, P3 and P4) per horse were evaluated similarly. The count of loci with one or more P variants for horses classified by P2 and P4 genotypes (no variants at these loci, variants at only P2, variants at only P4 or variants at both P2 and P4) was similarly compared. Additionally, to evaluate potential quantitative effects of these loci, multiple logistic regression was performed within each breed (WB and AR), using PSSM2 and/or MFM as the dependent binary variable and including sex (as binary), age (as continuous) and P2, P3A and P4 genotypes (as continuous; defined as 0 = homozygous reference, 1 = heterozygous and 2 = homozygous alternate allele). Lastly, the allele frequencies at each locus were calculated within each breed.

The odds ratio, sensitivity, specificity and positive and negative predictive values of the genetic variants were determined. For multiple logistic regression, area under the receiver operator curve (ROC AUC) was generated and log-likelihood ratios were used for hypothesis testing within each breed. Statistics were performed using commercially available software GraphPad Prism (version 8) and R (version 3.6.3).^26^ Data are presented as mean and standard deviation; *P* was set at < .05.

## 3 | RESULTS

### 3.1 | Horses

There were 54 control and 68 PSSM2/MFM-WB horses (Table 2) with information for horses classified as PSSM2 or MFM separately provided in Table S3. Age was not normally distributed and after log transformation there was no difference in age between control-WB and PSSM2/MFM-WB (*P* = .2). Thirty control-AR were available for comparison to 30 PSSM2/MFM-AR with information for separate PSSM2 and MFM groups provided in Table S3. Due to overlap of PSSM2 and MFM diagnosis, results for some horses are presented in both groups (PSSM2 or MFM). Age was normally distributed and there was no significant difference in ages between control-AR and PSSM2/MFM-AR (*P* = .3).

### 3.2 | Genotype and allele frequencies

**FIGURE 1:**
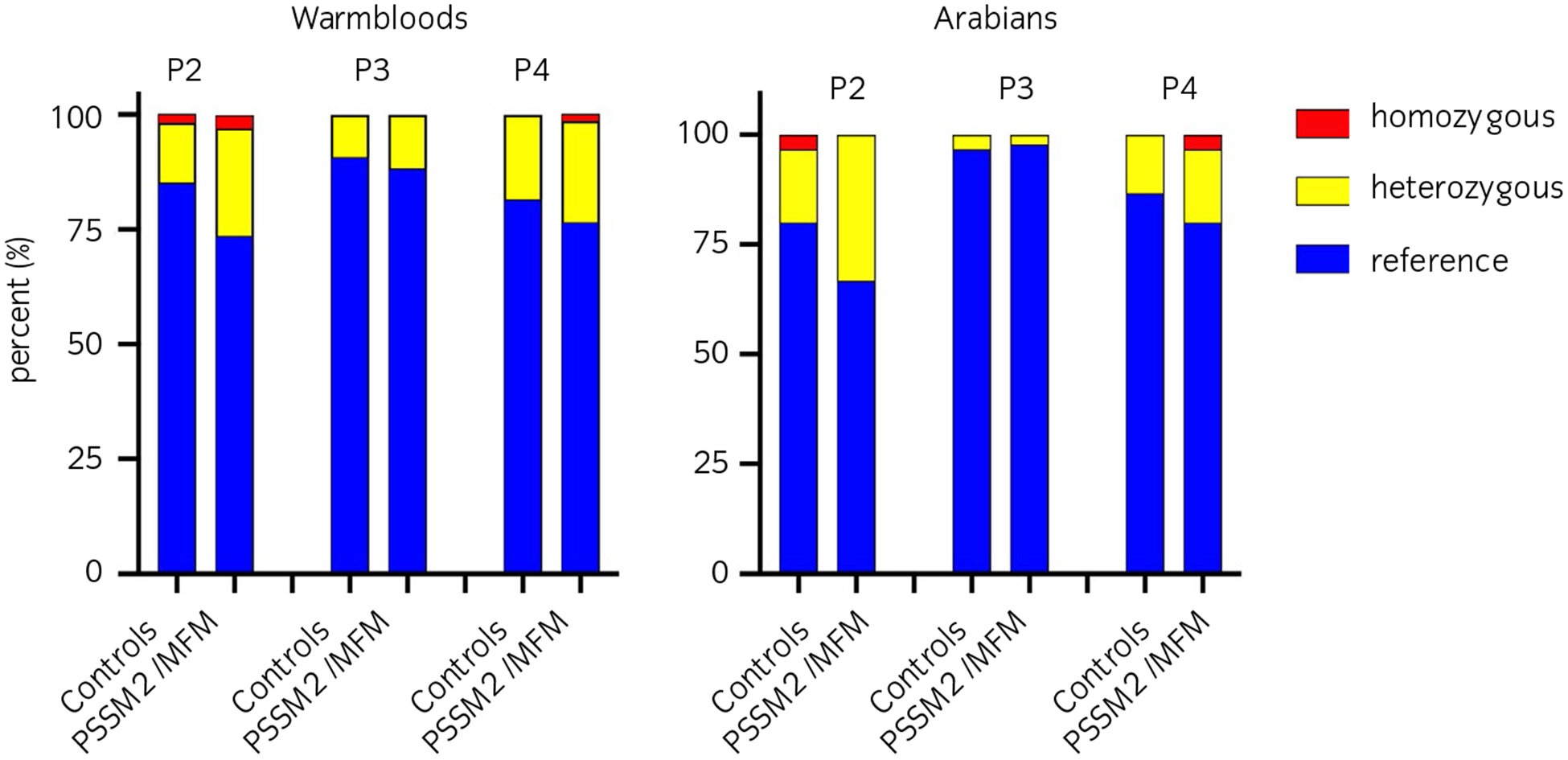
The percentage of horses homozygous for the reference allele (blue), heterozygous (yellow) or homozygous (red) for P2, P3a/P3b or P4 in Warmblood and Arabian horse controls and those diagnosed with PSSM2/MFM by muscle histopathology. 54 control-WB, 68 PSSM2/MFM-WB, 30 control-AR and 30 PSSM2/MFM-AR horses were analysed. There was no significant difference in the frequency of any of the P variants between control and PSSM2/MFM for either breed

All four loci were successfully genotyped in the WB and AR controls and MFM/PSSM2 horses; there were no missing data. There were no statistically significant differences in genotype (P2 *P* = .2, P3a/P3b *P* =.44, P4 *P* = .8) when comparing the subgroup of WB control horses with no clinical signs/shivers to the subgroup with overlapping clinical features of PSSM2/MFM (Figure S1A). There were no statistically significant differences in genotype or allele frequencies between all controls and horses classified as PSSM2/MFM, MFM or PSSM2 for any of the alternate alleles of P loci analysed by breed (Figure 1, Table 3, Table S4). The P2 variant occurred in 15% of control-WB and 27% of PSSM2/MFM-WB and 20% of control and 33% of PSSM2/MFM-AR with an overall frequency (cases and controls) of 0.119 in WB and 0.142 in AR. The P3a and P3b variants were in complete linkage disequilibrium, occurring in 9% of control-WB and 12% of PSSM2/MFM-WB and in 3% of AR regardless of phenotype. Overall frequencies for the P3 variant in each breed were 0.053 in WB and 0.017 in AR. The P4 variant was present in 19% of control-WB and 24% of PSSM2/MFM-WB as well as 13% of control-AR and 20% of PSSM2/MFM-AR with breed frequencies of 0.111 in WB and 0.092 in AR. There were no statistically significant differences in genotype frequencies between control-WB ≥7 years of age and PSSM2/MFM-WB for P2, P3a, P3b and P4 variants (P2 *P* = .07, P3a/P3b *P* > .99, P4 *P* = .4) (Figure S1B). Multiple logistic regression within WB and AR did not identify any significant contribution of loci to the phenotype (WB – ROC AUC = 0.64 ± 0.05, log-likelihood ratio = 5.86 (*P* =.32); AR – ROC AUC = 0.62 ± 0.07, log-likelihood ratio = 3.88 (*P* = .57)).

### 3.3 | Total number of variant alleles and loci

**FIGURE 2:**
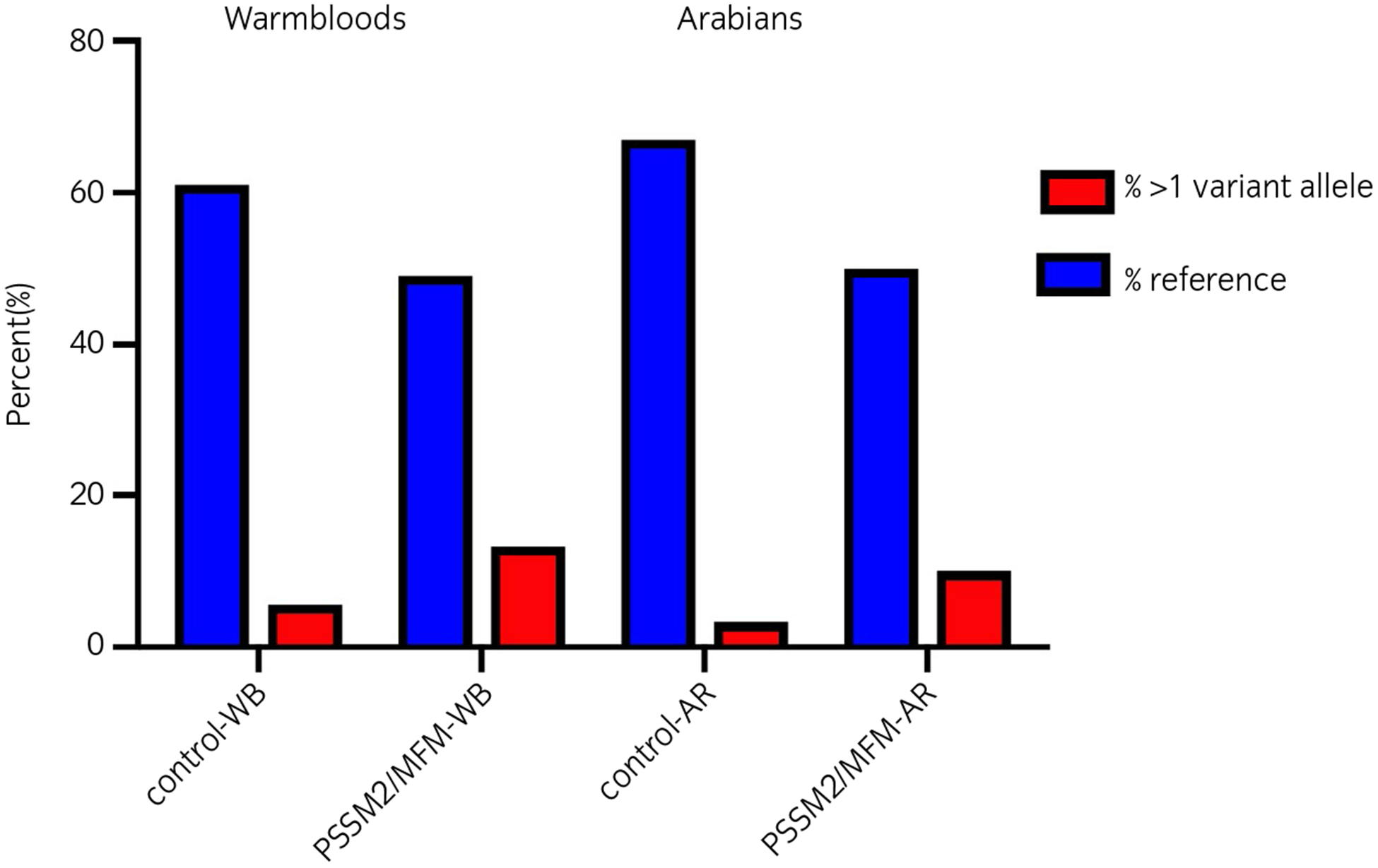
The percentage of control and PSSM2/MFM Warmblood (WB) and Arabian (AR) horses that had more than one P2, P3a/P3b or P4 variant alleles. There were no significant differences in the frequency of horses with none, one or more than one variant alleles between control and PSSM2/MFM horses by breed

There were no significant differences in the total number of P variants or in the number of P loci with at least one variant allele for any locus between control and either PSSM2/MFM or MFM or PSSM2 horses within breed (Figure 2, Table 4, Table S5). The percentage of horses with more than one variant allele across all P loci was 5.6% for control-WB and 13.2% for PSSM2/MFM-WB and 3.3% for control-AR and 10% for PSSM2/MFM-AR. The developers of the test have proposed that horses with P variants at both P2 and P4 are more likely to have severe signs of PSSM2 (http://equiseq.com/blog/p2-p4-linkage). We found that the frequency of PSSM2 horses with a variant at both P2 and P4 was less than 7% in both WB and AR (data not shown) and that both P2 and P4 variants were present in control-WB. PSSM2 horses did not have significantly different frequencies of P variants at both the P2 and P4 loci compared to controls (*P* = .763).

### 3.4 | Sensitivity of commercial genetic test variants

The sensitivity for testing ranged from 0.03 (P3 in the WB) to a maximum of 0.33 (P2 in the WB) (Table 5, Table S6). Odds ratios ranged from 0.7 (P4 in the WB) to 2.07 (P2 in the AR); however, all calculated confidence intervals included 1 and were therefore not significant.

### 3.5 | Allele frequencies in public databases

Genotype data were available for the P loci from 107 modern, 85 early domestic, 1 ancient and 12 Przewalski horses (N = 205 total; Table S2). Two samples noted as ‘ancient’ in the SRA were instead classified as modern horses due to the reported age of each sample (between 89 and 267 years old).^27^ Genotyping rate for the 205 horses ranged from 0.58 (P3b) to 0.82 (P2). The P2 variant was present at a frequency of 0.177 in the modern horses, and 0.176 in the early domestic horses; its frequency was 0.333 in 18 genotyped samples from 3501 to 5500 years ago. The P2 variant was also present in five of the 12 Przewalski horses. A third nucleotide (C), predicted to result in the substitution of serine with alanine, was identified in three Franches-Montagnes (two heterozygous and one homozygous; frequency = 0.02). Across all 168 samples in the SRA database with a P2 genotype, 32% possessed a P variant (43 heterozygotes/11 homozygotes). Within the public database, the P3a and P3b variants were not in complete linkage disequilibrium and found at frequencies of 0.09 and 0.06, respectively, in the modern horses. The P3a variant was identified as early as 5,500 years ago and had a frequency of 0.061 in the early domestic horses. The P3b variant dated to over 1900 years and was present at a frequency of 0.047 in early domestic horses. The variant alleles for P2 and P3 were found in horses sampled in Central Europe (Estonia), the Middle East (Turkey) and Eastern Asia (Mongolia), among other locations across Eurasia. The P4 variant was present in 21% of the modern horses from the database with the allele frequency of 0.135. The P4 variant was found in the one horse classified as early domestic (dating to 467 years before present), accounting for an allele frequency of 0.008 in the early domestic horses; this horse did not have any alternate alleles at the other P2, P3a and P3b loci.

## 4 | DISCUSSION

The lack of regulatory requirements for commercial genetic tests for horses makes it particularly important to report their diagnostic accuracy in peer-reviewed publications. Our analysis of commercially offered genetics tests for PSSM2 or MFM used the gold standard of phenotyping via histopathology. We found no association between any of the test variants, or combinations of test variants, and the presence of either PSSM2/MFM considered as one diagnosis, or when PSSM2 or MFM were considered separately in AR or WB. The sensitivity of the genetic tests, or true positive rate, was low at <33% and all confidence intervals for odds ratios included 1, indicating that the variants neither increase nor decrease the odds of disease. Healthy control horses were as likely to possess a P2, P3 or P4 variant as a horse with PSSM2 or MFM. Thus, we find no supporting evidence that the P2, P3 or P4 variants are themselves causative or diagnostic of a myopathy in the horse.

We found that P2, P3a and P3ba variants have been present in equids long before breed formation. Notably, the identification of the P2 variant in Przewalski horses, which are not direct ancestors of modern horses, dates the origin of the P2 variant to before the divergence of these taxa, estimated at 35 000 to 55 000 years ago.^28,29^ All four P variants identified in horses dated to before modern selection practices were established and were additionally widely distributed across the breeds studied. Thus, the use of the P variant genetic tests in selection decisions, prepurchase examination or diagnosis of a myopathy is not supported by the findings of the current study based on their presence in WB and AR regardless of phenotype and their presence in modern, early domestic and Przewalski horses.

The importance of assessing the diagnostic value of variants in the equine genome is highlighted by the fact that variants in genetic sequence, even those which change gene or protein function, are common across species. For example, a study of 88 horses from diverse breeds found that a single horse, on average, has over 1.8 million homozygous and over 3.9 million heterozygous SNP variants compared to the reference genome; of these variants, more than 400,000 were predicted to have protein-changing effects.^30^ Furthermore, RNA-seq analysis of muscle from eight WB MFM and eight control WB horses found 26 missense variants, including P2, P3a and P3b, in 16 genes known to cause MFM or MFM-like diseases in humans; none of the variants, however, were associated with the MFM phenotype in the eight WB horses.^31^ The relative ease with which genomes can be sequenced using high-throughput next-generation sequencing and the resultant identification of numerous variants in the genome underscore the importance of validating associations between a discovered variant and the gold standard for diagnosis of the associated disease.

The P2 variant occurs in the gene *MYOT* and is predicted to cause an amino acid substitution of a neutral proline for a neutral polar serine in the sarcomeric Z-disc protein myotilin; this substitution is predicted to be tolerated by SIFT analyses. Mutations in *MYOT* are validated causes of MFM in humans; however, the validated mutations impact other regions of the myotilin gene than P2.^32,33,34^ In AR, the P2 variant was present in 20% of healthy Arabians and absent in 66% of AR with PSSM2/MFM. In WB, the P2 variant was present in 15% of healthy WB and absent in 73% PSSM2/MFM horses. In addition, the P2 variant was found in horses of diverse breeds, lesser managed, ‘landrace’ breeds (eg Yakutian and Mongolian), early domestic horses and the Przewalski horse. Thus, the P2 variant appears to be a common variant long present in equids. Common variants are not necessarily benign; however, if a moderately frequent variant itself had a notable impact on phenotype, it would be unlikely to be perpetuated across such a time frame. These lines of evidence—its presence across evolutionary time, similar frequency in PSSM2/MFM horses and controls and lack of a predicted or demonstrated impact on gene function—support the conclusion that the P2 variant cannot be accurately used to diagnose PSSM2/MFM.

The P3a variant in *FLNC* is predicted to cause a substitution of the acidic amino acid glutamic acid annotated in the reference genome with the basic amino acid lysine and the P3b variant substitution of a neutral alanine with threonine. With the exception of P3b in one isoform of *FLNC*, both variants are predicted to be tolerated. Mutations in other sites in *FLNC*, which encode the Z-disc protein filamin C, have been shown to cause MFM in humans.^14,35^ The P3 variants, in complete linkage disequilibrium in these samples, were at low frequency in PSSM2/MFM, PSSM2 and MFM-WB and AR horses (< 12% of horses with a variant allele) with no difference in frequency compared to control horses. The very low sensitivity of the P3 variants for predicting PSSM2/MFM indicates that these P3 variants cannot accurately be used to diagnose PSSM2 or MFM.

The P4 variant is predicted to cause a deleterious substitution of serine with leucine in Z-disc protein myozenin 3 expressed in fast-twitch muscle fibres. A very similar protein, myozenin 1, is also expressed in fast-twitch myofibres, has a similar function and could possibly compensate for a myozenin 3 deficiency.^36^ Mutations in *MYOZ3* have not been reported to cause MFM or PSSM2 in any species; however, mutations in *MYOZ2*, solely expressed in cardiac muscle, cause a cardiomyopathy characterised by myofibrillar disarray.^36^ Only 24% of PSSM2/MFM-WB and 20% of PSSM2/MFM-AR possessed the P4 variant and the frequency of P4 in PSSM2/MFM was not statistically different than control horses. Similar to the results from the other loci, there is no indication that the P4 variant itself is predictive of, or causes, PSSM2/MFM.

It has been argued that a combination of the P variants is required for the expression of PSSM2 or MFM. The developers of the test have proposed that horses with variants at both P2 and P4 are more likely to have severe signs of PSSM2 (http://equiseq.com/blog/p2-p4-linkage). To investigate the possible role of multiple variants, we compared the number of horses with zero, one or more than one variant alleles or loci between control and PSSM2/MFM, PSSM2 or MFM, as well as the number of horses with at least one alternate allele at both the P2 and P4 loci. Again, there were no differences between controls and either MFM/PSSM2 or PSSM2 or MFM groups in the number of horses with multiple variant alleles, multiple variant loci or variant alleles at both P2 and P4. Furthermore, logistic regression failed to identify any significant contribution of loci to the PSSM2/MFM phenotype. Thus, a combination of P variants is not clinically useful to diagnose either MFM or PSSM2. It is possible that the alternative alleles at the P loci could explain a small proportion of variance in phenotype of MFM/PSSM2 horses, as would be expected in the case of a complex trait. The results of our study, however, do not support their use as diagnostic markers of either PSSM2 or MFM.

No functional evidence has demonstrated that any of the P variant alleles impact, positively or negatively, the health and fitness of the horse. The presence of three nucleotide variants at the P2 locus suggests that conservation of this residue is likely not functionally important. If any of these variants did confer a disadvantage to health and fitness, as would be expected for a horse diagnosed with MFM or PSSM2, they would be unlikely to have been perpetuated through domestication as well as maintained at relatively high frequency in a diversity of breeds.

One concern in the present study was the definition of control horses. For AR horses, control horses had a well-known history with no reported episodes of muscle disease and no evidence of muscle histopathology. Using this control group, there was no evidence to support any of the P variants being associated with a diagnosis of PSSM2/MFM by muscle histopathology. For WB horses, there were not enough muscle biopsy samples from healthy WB (N = 11) to conduct a similar analysis. We elected to define control WB horses as those with no evidence of muscle histopathology. Sixteen of these horses likely had a neurologic disorder with clinical signs of stringhalt/shivers.^37^ Twenty-seven horses had a wide variety of common clinical signs in athletic horses ranging from lameness to poor performance to sore muscles. To address the concern that some of the control WB could have had PSSM2/MFM that was not detected by muscle biopsy, we subdivided the WB control group into those with no overlapping signs of PSSM2/MFM and those with potential signs of PSSM2/MFM, such as muscle soreness, rhabdomyolysis, lameness/gait abnormalities and poor performance. There were no statistically significant differences in genotypes for any of the P variants between the two control WB subgroups. There was a tendency towards a higher frequency of the P2 variant in WB controls with signs that overlap PSSM2/MFM; however, in light of the fact that 73% of the PSSM2/MFM horses diagnosed by histopathology lacked the P2 variant, this variant is not a useful diagnostic marker for PSSM2/MFM. The lack of any statistical association between any P variant and PSSM2/MFM in either the well-defined control and myopathic AR horses or the control and myopathic WB horses thus indicates that the P variants are not useful as diagnostic tests for PSSM2/MFM.

Because it has not yet been established whether PSSM2 is an earlier manifestation of MFM in WB and AR, we performed our analyses considering them as one disease as well as considering them as separate diagnoses. Regardless, we found no significant association between any of the P variants and the disease phenotype. MFM is considered an adult-onset disease often evident in WB 7 years of age or older.^9^ In our study, ages were similar between control and PSSM2/MFM-WB and PSSM2/MFM-AR horses. Nevertheless, we performed an additional analysis whereby the WB control horses were stratified by age and compared using only WB 7 years of age or older to PSSM2/MFM-WB. No significant differences in genotypes were found. Although not expected, even if PSSM2/MFM was to develop in some of the control horses used in this study at a later age, the low frequency of the variants at these loci in horses that were diagnosed with PSSM2 and/or MFM, and thus their low PPVs, again demonstrates that these variants are not predictive of PSSM2/MFM.

It could be argued that PAS or desmin stains are not the most accurate way to diagnose PSSM2 or MFM respectively. The presence of aggregates of PAS or aggregates of desmin-positive material, combined with supportive ultrastructural features, however, was the means by which these diseases were defined. Protein aggregates of desmin and several other myofibrillar and cytoplasmic proteins are a hallmark of MFM in humans that is caused by a wide variety of mutations in a wide variety of genes.^11,12,13,14,38^ Horses with desmin aggregates would likely be the most extreme phenotype if MFM represents a later stage of PSSM2 in WB and AR and therefore, MFM horses would be expected to possess one or more P variants if they were predictive of disease. We found, however, that <20% of MFM horses possessed one P variant and <22% possessed two or more P variants indicating that the presence of variants alleles at multiple variant loci is not associated with a diagnosis of PSSM2 or MFM. In order to prevent any bias in interpretation, data were carefully evaluated, analysed and interpreted by researchers at three Universities (University of Nebraska, Lincoln, University of California, Davis and Oregon State University) independent of the laboratory performing the histopathological diagnoses (Michigan State University). Thus, independent analysis of the genotypes of horses diagnosed with PSSM2 or MFM using the current gold standards for phenotyping confirmed that there was no association between the P2, P3a, P3b and P4 loci and the disease phenotype.

In conclusion, the P2, P3 and P4 variants have been perpetuated across the timeline of domestication and breed formation in horses. Our results indicate that the variants used in commercial genetic tests are not predictive of the presence or absence of PSSM2 or MFM in AR and WB diagnosed by the well-established criteria of clinical signs and histopathology. Given the frequencies identified in these samples, by chance alone 29% of WB and 25% of AR will have at least one P variant regardless of their phenotype. While a noninvasive means to detect susceptibility to PSSM2 or MFM at an early age is highly desirable, at present, our study findings indicate that a careful history, physical examination, serum creatine kinase activity, PSSM1 genetic test and, in the absence of PSSM1, muscle histopathology represent more accurate and validated means to differentiate the many causes of exertional myopathies in horses.^39,40^

## Supplementary Material

Supp Table 1

Supp Table 2

Supp Fig 1

Supp Table 3

Supp Table 4

Supp Table 5

Supp Table 6

Infographic

## Figures and Tables

**TABLE 1:** The gene, Ensembl transcript ID used for annotation, chromosomal location, cDNA sequence variation and amino acid substitution for the P2, P3a, P3b and P4 variants with reference to EquCab3

		Annotation				
Variant	Gene/Transcript ID	gDNA	cDNA	Protein	Variant ID	SIFT prediction
P2	Myotilin (*MYOT*) ENSECAT00000022238.3	chr14:g.37818823A>G	c.697T>C	p.Ser233Pro	rs1138656462	0.18 (tolerated)
P3a	Filamin C (*FLNC*) ENSECAT00000076596.1	chr4:g.83837774G>A	c.2971G>A	p.Glu991Lys	rs1139799323	0.06–0.08 (tolerated)
P3b	Filamin C (*FLNC*) ENSECAT00000076596.1	chr4:g.83840299G>A	c.4333G>A	p.Ala1445Thr	rs1142918816	0.04 (deleterious) – 0.07 (tolerated), isoform dependent
P4	Myozenin 3 (*MYOZ3*) ENSECAT00000015783.3	chr14:g.26710261G>A	c.125C>T	p.Ser42Leu	rs1142544043	0 (deleterious)

**TABLE 2:** The age (mean and SD range), sex, glycogen synthase 1 (*GYS1*) mutation status, muscle biopsied, number of horses with PAS-positive aggregates and the number with four or more fibres with desmin aggregates for Warmblood (WB) and Arabian (AR) horse controls and those diagnosed with type 2 polysaccharide storage myopathy and myofibrillar myopathy

	Warmblood		Arabian	
	Control	PSSM2/MFM	Control	PSSM2/MFM
N	54	68	30	30
Age (y) Range	8.6 ± 4.3 2–24	9.7 ± 4.6 3–28	12.4 ± 5.9 4–23	13.9 ± 5.7 4–29
Sex	20 f, 33 mc, 1 s	23 f, 42 mc, 2 s, 1 un	22 f, 8 mc	18 f, 12 mc
*GYS1* mutation	neg	neg	nd	13 neg, 17 nd
Muscle biopsied	29 glut, 34 sm	18 glut 50 sm	30 glut	17 glut, 13 sm
Horses with PAS aggregates	0	55	0	18
Horses with desmin aggregates	0	37	0	30

Abbreviations: AR, Arabian; f, female; Glut, gluteus medius; GYS1, glycogen synthase 1; mc, male castrate; MFM, Myofibrillar Myopath; nd, not done; neg, negative; PAS, periodic acid-Schiff’s; PSSM2, Polysaccharide storage myopathy type 2; s, stallion; Sm, semimembranosus; un, unknown; WB, Warmblood.

**TABLE 3:** The number of horses homozygous for the reference allele, heterozygous or homozygous for each P variant by phenotype and breed as well as the percentage of horses in each classification possessing each P variant and the variant allele frequencies

	N	Homozygous reference	Heterozygous	Homozygous Variant	% Horses Heterozygous or Homozygous Variant	Variant Allele Frequency	*P*-value	
							Genotypic	Dominant
P2: *MYOT-* rs1138656462								
Control-WB	54	46	7	1	15%	0.08		
PSSM2/MFM-WB	68	50	16	2	27%	0.15	.32	.23
Control-AR	30	24	5	1	20%	0.12		
PSSM2/MFM-AR	30	20	10	0	33%	0.17	.82	1.00
P3a/P3b: *FLNC*- rs1139799323/ *FLNC* - rs1142918816								
Control-WB	54	49	5	0	9%	0.05		
PSSM2/MFM-WB	68	60	8	0	12%	0.06	1.00	1.00
Control-AR	30	29	1	0	3%	0.02		
PSSM2/MFM-AR	30	29	1	0	3%	0.02	1.00	1.00
P4: *MYOZ3* - rs1142544043								
Control-WB	54	44	10	0	19%	0.09		
PSSM2/MFM-WB	68	52	15	1	24%	0.13	1.00	.82
Control-AR	30	26	4	0	13%	0.07		
PSSM2/MFM-AR	30	24	5	1	20%	0.12	.61	1.00

*Note: P*-values are given for comparisons of the prevalence of the variant in PSSM2/MFM vs. controls using genotypic or dominant models. The predicted amino acid substitutions were based upon the variant positions and Ensembl transcript ENSECAT00000076596.1. There were no significant differences in the prevalence of the variants or allele frequencies between control and PSSM2/MFM horses of either breed.

Abbreviations: MFM, Myofibrillar Myopathy; PSSM2, Polysaccharide storage myopathy type 2.

**TABLE 4:** The total number of control and PSSM2/MFM horses of each breed and counts within each denoting the number of horses with zero P variant alleles/loci, one P variant allele or locus or > 1 P variant alleles/loci

	N	No Variants	One Variant Allele/Locus	> 1 Variant Alleles/Loci	% >1 P Variant Allele/Loci	*P*-value (allele/locus)
Control-WB	54	33	18/19	3/2	5.6/3.7	
PSSM2/MFM-WB	68	33	26/28	9/7	13.2/10.3	0.26/0.23
Control-AR	30	20	9/9	1/1	3.3/3.3	
PSSM2/MFM-AR	30	15	12/13	3/2	10.0/6.7	0.35/0.45

*Note: P*-values are given for comparisons of the number of horses with none, one or >1 P variant allele/loci between control and PSSM2/MFM horses by breed.

Abbreviations: AR, Arabian; MFM, Myofibrillar Myopathy; PSSM2, Polysaccharide storage myopathy type 2; WB, Warmblood.

**TABLE 5:** The odds ratio (OR), sensitivity, specificity, positive predictive value (PPV) and negative predictive value (NPV) for the P2, P3 and P4 variants in PSSM2/MFM horses by breed. P3a and P3b were in complete linkage disequilibrium

	Variant	OR		Sensitivity		Specificity		PPV		NPV	
			CI		CI		CI		CI		CI
PSSM2/MFM-WB	P2	2.07	0.82–5.12	0.27	0.18–0.38	0.85	0.73–0.92	0.69	0.50–0.84	0.48	0.38–0.58
	P3	1.31	0.43–3.75	0.12	0.06–0.23	0.91	0.80–0.96	0.62	0.36–0.82	0.45	0.36–0.54
	P4	1.35	0.56–3.36	0.24	0.15–0.34	0.82	0.69–0.90	0.62	0.43–0.78	0.46	0.36–0.56
PSSM2/MFM-AR	P2	2.00	0.60–7.05	0.33	0.19–0.51	0.80	0.63–0.90	0.63	0.39–0.82	0.55	0.40–0.68
	P3	1.00	0.05–19.6	0.03	0.002–0.17	0.97	0.83–0.99	0.50	0.03–0.98	0.50	0.38–0.63
	P4	1.62	0.45–5.56	0.20	0.09–0.37	0.87	0.70–0.95	0.60	0.31–0.83	0.52	0.39–0.65

*Note*: None of the calculated values reached statistical significance (*P* < .05).

Abbreviations: AR, Arabian; MFM, Myofibrillar Myopathy; NPV, negative predictive value; PPV, positive predictive value; PSSM2, Polysaccharide storage myopathy type 2; WB, Warmblood.
