# Supplementary material for: Commercial genetic testing for type 2 polysaccharide storage myopathy and myofibrillar myopathy does not correspond to a histopathological diagnosis
Source: Equine Vet J. Author manuscript; Available in PMC 2021 Jul 1. (PMC7937766; doi:10.1111/evj.13345)
Supplement: Supp Table 1 [file NIHMS1661435-supplement-Supp_Table_1.pdf]

Equine Veterinary Journal BEVA

Table S1: P variant coordinates, SNP identifiers, and primers utilised for genotyping (EquCab 2) by pyrosequencing.

SNP	Gene	Chromo-some	Coordinate	SNP ID and polymorphism (coding strand)	Fwd Primer (5'-3')	Rev Primer (5'-3')	Sequencing Primer (5'-3')
P2	*MYOT*	14	38519183	rs1138656462<br>(T/C)	CTGGCACTTGAA TGAAACGA	TGATAATTTTCCGCA TGGTGA	ACATCTCCCCTTGAAG
P3a	*FLNC*	4	83736244	rs1139799323<br>(G/A)	GAGCCCACCTAT TTCACCG	CAGGCCCCGTCTCC TACT	TTCACCGTGGACTGC
P3b	*FLNC*	4	83738769	rs1142918816<br>(G/A)	CTTCCCCGGCAC CTACACTATT	CCCGAGACCTTGAC TCCACT	CGCGTCCATGTGCAG
P4	*MYOZ3*	14	27399222	rs1142544043<br>(C/T)	CGTGCCCCAGGA CCTGAT	GCTGGCTGGCTGCA AACT	GATGATGGAAGAGCTGT
