# Supplementary material for: Commercial genetic testing for type 2 polysaccharide storage myopathy and myofibrillar myopathy does not correspond to a histopathological diagnosis
Source: Equine Vet J. Author manuscript; Available in PMC 2021 Jul 1. (PMC7937766; doi:10.1111/evj.13345)
Supplement: Supp Table 2 [file NIHMS1661435-supplement-Supp_Table_2.pdf]

Equine Veterinary Journal BEVA

**Table S2:** Genomes surveyed from the NCBI SRA from which one or more P variant genotypes were available. Genotypes matching the reference allele are denoted by a period. nc indicates the genotype was not called due to failure to meet quality criteria (e.g. minimum 5 reads). Samples are classified as modern, early domestic (400-500 years old), or ancient with breed, species, or sample origin noted when available.

| | A | B | C | D | E | F | G |
|---|---|---|---|---|---|---|---|
| 3 | | **P2** | **P3b** | **P3a** | **P4** | | |
| 4 | **RunID/Ref** | **A** | **G** | **G** | **G** | **Classification** | **Breed/Origin** |
| 5 | ERR3628174 | nc | nc | . | nc | Modern | Franches-Montagnes |
| 6 | ERR979788 | AG | nc | nc | nc | Modern | Franches-Montagnes |
| 7 | ERR2731061 | nc | nc | nc | . | Modern | Holsteiner |
| 8 | ERR863167 | . | nc | nc | nc | Modern | Icelandic Horse |
| 9 | ERR793393 | . | nc | nc | nc | Modern | Icelandic Horse |
| 10 | ERR3628173 | nc | nc | nc | . | Modern | unknown |
| 11 | ERR1527960 | AG | nc | nc | . | Modern | Franches-Montagnes |
| 12 | ERR1527953 | CC | nc | nc | GA | Modern | Franches-Montagnes |
| 13 | ERR1527954 | GG | nc | nc | . | Modern | Franches-Montagnes |
| 14 | ERR1527957 | . | nc | nc | AA | Modern | Franches-Montagnes |
| 15 | ERR2179540 | . | nc | nc | . | Modern | Franches-Montagnes |
| 16 | ERR979790 | AG | nc | nc | . | Modern | Franches-Montagnes |
| 17 | ERR1545182 | . | . | nc | nc | Modern | German WarmBlood |
| 18 | ERR1545185 | . | nc | nc | . | Modern | German WarmBlood |
| 19 | ERR1545180 | . | nc | nc | . | Modern | German WarmBlood |
| 20 | ERR1527966 | . | nc | nc | . | Modern | Haflinger |
| 21 | ERR2179547 | . | . | nc | nc | Modern | Holsteiner |
| 22 | ERR979800 | . | nc | nc | . | Modern | Quart Horse |
| 23 | ERR3628171 | AG | . | nc | nc | Modern | unknown |
| 24 | ERR1305962 | . | nc | . | . | Modern | American Paint Horse |
| 25 | ERR1527967 | . | . | nc | . | Modern | Dutch Warmblood |
| 26 | ERR979794 | . | . | nc | . | Modern | Franches-Montagnes |
| 27 | ERR979793 | GG | . | . | nc | Modern | Franches-Montagnes |
| 28 | ERR979785 | AG | . | nc | . | Modern | Franches-Montagnes |
| 29 | ERR1527952 | . | . | nc | . | Modern | Franches-Montagnes |
| 30 | ERR979795 | AG | . | nc | . | Modern | Franches-Montagnes |
| 31 | ERR1527961 | AG | . | nc | GA | Modern | Franches-Montagnes |
| 32 | ERR979784 | AG | GA | nc | . | Modern | Franches-Montagnes |
| 33 | ERR1527959 | . | . | nc | GA | Modern | Franches-Montagnes |
| 34 | ERR1527965 | AG | AA | nc | AA | Modern | Franches-Montagnes |
| 35 | ERR1527964 | . | AA | nc | GA | Modern | Franches-Montagnes |
| 36 | ERR466186 | . | . | nc | . | Modern | Franches-Montagnes |
| 37 | ERR2179541 | AC | . | nc | GA | Modern | Franches-Montagnes |
| 38 | ERR1545179 | . | . | nc | . | Modern | German WarmBlood |
| 39 | ERR1545184 | . | . | nc | GA | Modern | German WarmBlood |
| 40 | ERR1545187 | . | . | nc | . | Modern | German WarmBlood |
| 41 | ERR979798 | . | . | . | nc | Modern | Quarter Horse |
| 42 | ERR979797 | GG | . | nc | AA | Modern | Quarter Horse |
| 43 | ERR979802 | . | . | nc | . | Modern | Standardbred |
| 44 | ERR979801 | AG | . | nc | . | Modern | Standardbred |
| 45 | ERR1735862 | . | nc | . | . | Modern | Thoroughbred |
| 46 | ERR979796 | . | nc | . | . | Modern | unknown |
| 47 | ERR1021816 | . | . | nc | . | Modern | Yakutian Horse |
| 48 | ERR1021818 | . | . | nc | . | Modern | Yakutian Horse |
| 49 | ERR1021823 | . | . | nc | . | Modern | Yakutian Horse |

| | A | B | C | D | E | F | G |
|---|---|---|---|---|---|---|---|
| 50 | ERR1021824 | . | GA | nc | . | Modern | Yakutian Horse |
| 51 | ERR1021819 | . | GA | nc | . | Modern | Yakutian Horse |
| 52 | ERR1527950 | . | . | . | . | Modern | Akhal-Teke |
| 53 | ERR1527948 | . | . | . | . | Modern | Akhal-Teke |
| 54 | ERR1527949 | . | . | . | . | Modern | Akhal-Teke |
| 55 | ERR1527947 | . | . | . | . | Modern | Akhal-Teke |
| 56 | ERR1305964 | . | . | . | . | Modern | American Paint Horse |
| 57 | ERR1305963 | . | . | . | . | Modern | American Paint Horse |
| 58 | ERR2179544 | GG | . | . | . | Modern | Arabian |
| 59 | ERR2179551 | AG | . | . | . | Modern | Arabian |
| 60 | ERR1527951 | . | . | . | . | Modern | Arabian |
| 61 | ERR1527971 | . | . | . | GA | Modern | Franches-Montagnes |
| 62 | ERR1527955 | . | . | . | AA | Modern | Franches-Montagnes |
| 63 | ERR979792 | . | . | . | . | Modern | Franches-Montagnes |
| 64 | ERR979789 | AG | . | . | . | Modern | Franches-Montagnes |
| 65 | ERR1527962 | AG | . | GA | . | Modern | Franches-Montagnes |
| 66 | ERR979787 | . | . | . | GA | Modern | Franches-Montagnes |
| 67 | ERR979791 | AC | . | . | . | Modern | Franches-Montagnes |
| 68 | ERR979786 | AG | GA | GA | GA | Modern | Franches-Montagnes |
| 69 | ERR1527963 | AG | GA | GA | . | Modern | Franches-Montagnes |
| 70 | ERR1527956 | AG | . | . | . | Modern | Franches-Montagnes |
| 71 | ERR1527958 | AG | . | . | GA | Modern | Franches-Montagnes |
| 72 | ERR2179545 | . | . | . | . | Modern | German Riding Pony |
| 73 | ERR2179542 | . | . | . | . | Modern | German Riding Pony |
| 74 | ERR1545183 | AG | . | . | . | Modern | German Warmblood |
| 75 | ERR1545178 | . | . | . | GA | Modern | German Warmblood |
| 76 | ERR1545189 | . | . | . | . | Modern | German Warmblood |
| 77 | ERR1545190 | . | . | . | . | Modern | German Warmblood |
| 78 | ERR1545186 | . | . | . | . | Modern | German Warmblood |
| 79 | ERR1545181 | . | . | . | . | Modern | German Warmblood |
| 80 | ERR1545188 | . | . | . | . | Modern | German Warmblood |
| 81 | ERR2179553 | . | . | . | . | Modern | Haflinger |
| 82 | ERR2179554 | . | . | . | . | Modern | Haflinger |
| 83 | ERR2179555 | AG | . | . | . | Modern | Haflinger |
| 84 | ERR2179549 | AG | . | . | . | Modern | Hannoveraner |
| 85 | ERR2731057 | . | . | . | . | Modern | Icelandic Horse |
| 86 | ERR982704 | AG | . | . | . | Modern | Mongolian |
| 87 | ERR2179546 | AG | . | . | AA | Modern | Morgan Horse |
| 88 | ERR2179552 | . | . | . | . | Modern | Noriker |
| 89 | ERR2179548 | . | . | . | . | Modern | Oldenburg Horse |
| 90 | ERR982707 | AG | . | . | . | Modern | Polish Warmblood |
| 91 | ERR979799 | AG | . | . | . | Modern | Quarter Horse |
| 92 | ERR1527969 | . | . | . | . | Modern | Quarter Horse |
| 93 | ERR1527970 | . | . | . | . | Modern | Quarter Horse |
| 94 | ERR1527968 | . | . | . | . | Modern | Quarter Horse |
| 95 | ERR868004 | . | . | . | . | Modern | Shetland Pony |
| 96 | ERR868003 | . | . | . | . | Modern | Shetland Pony |

| | A | B | C | D | E | F | G |
|---|---|---|---|---|---|---|---|
| 97 | ERR979803 | AG | . | . | . | Modern | Standardbred |
| 98 | ERR1512897 | . | . | . | GA | Modern | Standardbred |
| 99 | ERR2179556 | . | . | . | . | Modern | Swiss Warmblood |
| 100 | ERR1527972 | . | . | . | . | Modern | Swiss Warmblood |
| 101 | ERR2203766 | . | GA | GA | GA | Modern | Trakehner |
| 102 | ERR1306526 | . | GA | GA | . | Modern | UK Warmblood (>50% Quarter Horse) |
| 103 | ERR1305961 | . | AA | AA | GA | Modern | UK Warmblood (>50% Quarter Horse) |
| 104 | ERR2179543 | . | . | . | AA | Modern | Welsh Pony |
| 105 | ERR2179550 | . | . | . | . | Modern | Welsh Pony |
| 106 | ERR1021822 | . | . | . | . | Modern | Yakutian Horse |
| 107 | ERR1021817 | . | . | . | . | Modern | Yakutian Horse |
| 108 | ERR1021821 | GG | . | . | . | Modern | Yakutian Horse |
| 109 | ERR1021820 | . | GA | GA | . | Modern | Yakutian Horse |
| 110 | ERR3225567 | nc | . | nc | nc | Modern | Thoroughbred |
| 111 | ERR3225630 | nc | GA | . | nc | Modern | UK |
| 112 | ERR3225467 | . | . | . | GA | Early Domestic (400-1500 years) | France |
| 113 | ERR3225602 | . | nc | . | . | Early Domestic (400-1500 years) | Mongolia |
| 114 | ERR3225600 | . | . | . | . | Early Domestic (400-1500 years) | Mongolia |
| 115 | ERR3225559 | nc | . | nc | nc | Early Domestic (400-1500 years) | Lithuania |
| 116 | ERR3225580 | AG | nc | nc | nc | Early Domestic (400-1500 years) | Estonia |
| 117 | ERR3225562 | nc | . | nc | . | Early Domestic (400-1500 years) | Lithuania |
| 118 | ERR3225639 | . | . | . | . | Early Domestic (400-1500 years) | Turkey |
| 119 | ERR3225570 | nc | . | . | . | Early Domestic (400-1500 years) | Croatia |
| 120 | ERR3225558 | nc | . | . | nc | Early Domestic (400-1500 years) | Lithuania |
| 121 | ERR2112789 | . | . | nc | . | Early Domestic (400-1500 years) | Kazakhstan |
| 122 | ERR3225483 | . | nc | . | nc | Early Domestic (400-1500 years) | Kyrgyzstan |
| 123 | ERR3225482 | . | GA | GA | nc | Early Domestic (400-1500 years) | Kyrgyzstan |
| 124 | ERR3225635 | nc | . | . | nc | Early Domestic (400-1500 years) | Turkey |
| 125 | ERR3225544 | . | . | . | . | Early Domestic (400-1500 years) | Mongolia |
| 126 | ERR3225655 | . | . | . | . | Early Domestic (400-1500 years) | Turkey |
| 127 | ERR3225553 | nc | nc | . | nc | Early Domestic (400-1500 years) | Germany |

| | A | B | C | D | E | F | G |
|---|---|---|---|---|---|---|---|
| 128 | ERR3225637 | . | . | . | . | Early Domestic (400-1500 years) | Turkey |
| 129 | ERR3225636 | . | . | nc | nc | Early Domestic (400-1500 years) | Turkey |
| 130 | ERR3225643 | . | nc | nc | nc | Early Domestic (400-1500 years) | Turkey |
| 131 | ERR3225658 | nc | nc | nc | . | Early Domestic (400-1500 years) | Turkey |
| 132 | ERR3225646 | nc | nc | . | nc | Early Domestic (400-1500 years) | Turkey |
| 133 | ERR3225648 | nc | nc | nc | . | Early Domestic (400-1500 years) | Turkey |
| 134 | ERR3225654 | . | . | . | . | Early Domestic (400-1500 years) | Turkey |
| 135 | ERR3225657 | AG | GA | AA | . | Early Domestic (400-1500 years) | Turkey |
| 136 | ERR3225593 | . | . | . | . | Early Domestic (1501-2500 years) | Iran |
| 137 | ERR3225647 | . | GA | nc | . | Early Domestic (1501-2500 years) | Turkey |
| 138 | ERR3225549 | AG | nc | nc | nc | Early Domestic (1501-2500 years) | Iran |
| 139 | ERR2112797 | . | . | nc | . | Early Domestic (1501-2500 years) | Turkey |
| 140 | ERR3225481 | nc | nc | nc | . | Early Domestic (1501-2500 years) | France |
| 141 | ERR3225640 | . | GA | nc | nc | Early Domestic (1501-2500 years) | Turkey |
| 142 | ERR3225552 | nc | . | nc | nc | Early Domestic (1501-2500 years) | France |
| 143 | ERR3225527 | . | nc | nc | nc | Early Domestic (1501-2500 years) | France |
| 144 | ERR3225530 | nc | . | nc | nc | Early Domestic (1501-2500 years) | Germany |
| 145 | ERR3225515 | nc | nc | nc | . | Early Domestic (1501-2500 years) | France |
| 146 | ERR3225504 | nc | nc | GA | . | Early Domestic (1501-2500 years) | France |
| 147 | ERR3225499 | . | . | . | . | Early Domestic (1501-2500 years) | France |

| | A | B | C | D | E | F | G |
|---|---|---|---|---|---|---|---|
| 148 | ERR2112790 | nc | . | nc | nc | Early Domestic (1501-2500 years) | Germany |
| 149 | ERR2112788 | AG | . | . | nc | Early Domestic (1501-2500 years) | Mongolia |
| 150 | ERR3225532 | nc | . | . | . | Early Domestic (1501-2500 years) | Mongolia |
| 151 | ERR3225534 | AG | . | nc | . | Early Domestic (1501-2500 years) | Mongolia |
| 152 | ERR3225531 | . | nc | . | . | Early Domestic (1501-2500 years) | Mongolia |
| 153 | ERR3225533 | . | nc | nc | nc | Early Domestic (1501-2500 years) | Mongolia |
| 154 | ERR3225535 | nc | . | . | nc | Early Domestic (1501-2500 years) | Mongolia |
| 155 | ERR3225586 | nc | . | nc | nc | Early Domestic (1501-2500 years) | France |
| 156 | ERR3225453 | nc | nc | nc | . | Early Domestic (1501-2500 years) | France |
| 157 | ERR3225451 | . | nc | nc | nc | Early Domestic (1501-2500 years) | France |
| 158 | ERR2112715 | . | . | nc | nc | Early Domestic (1501-2500 years) | France |
| 159 | ERR3225459 | nc | . | . | nc | Early Domestic (1501-2500 years) | Russia |
| 160 | ERR3225523 | nc | nc | . | nc | Early Domestic (2501-3500 years) | Spain |
| 161 | ERR3225579 | AG | GA | nc | . | Early Domestic (2501-3500 years) | Estonia |
| 162 | ERR2112796 | . | nc | . | . | Early Domestic (2501-3500 years) | Mongolia |
| 163 | ERR3225621 | AG | . | . | . | Early Domestic (2501-3500 years) | Mongolia |
| 164 | ERR3225614 | nc | . | nc | nc | Early Domestic (2501-3500 years) | Mongolia |

| | A | B | C | D | E | F | G |
|---|---|---|---|---|---|---|---|
| 165 | ERR3225617 | nc | . | nc | nc | Early Domestic (2501-3500 years) | Mongolia |
| 166 | ERR3225622 | nc | nc | . | nc | Early Domestic (2501-3500 years) | Mongolia |
| 167 | ERR3225620 | . | nc | nc | . | Early Domestic (2501-3500 years) | Mongolia |
| 168 | ERR3225615 | nc | . | nc | . | Early Domestic (2501-3500 years) | Mongolia |
| 169 | ERR3225623 | . | . | . | . | Early Domestic (2501-3500 years) | Mongolia |
| 170 | ERR3225619 | nc | . | . | . | Early Domestic (2501-3500 years) | Mongolia |
| 171 | ERR2112795 | . | . | . | . | Early Domestic (2501-3500 years) | Mongolia |
| 172 | ERR3225618 | . | . | . | . | Early Domestic (2501-3500 years) | Mongolia |
| 173 | ERR2112763 | nc | nc | nc | . | Early Domestic (2501-3500 years) | Russia |
| 174 | ERR3225465 | . | nc | . | nc | Early Domestic (2501-3500 years) | Russia |
| 175 | ERR3225541 | . | nc | nc | nc | Early Domestic (3501-5500 years) | Kazakhstan |
| 176 | ERR3225489 | . | nc | nc | nc | Early Domestic (3501-5500 years) | Spain |
| 177 | ERR3225490 | . | nc | nc | . | Early Domestic (3501-5500 years) | Spain |
| 178 | ERR2112765 | AG | . | . | . | Early Domestic (3501-5500 years) | Kazakhstan |
| 179 | ERR2112768 | . | nc | . | nc | Early Domestic (3501-5500 years) | Kazakhstan |
| 180 | ERR2112767 | AG | . | . | . | Early Domestic (3501-5500 years) | Kazakhstan |
| 181 | ERR2112766 | GG | nc | nc | . | Early Domestic (3501-5500 years) | Kazakhstan |

| | A | B | C | D | E | F | G |
|---|---|---|---|---|---|---|---|
| 182 | ERR2112764 | nc | . | . | . | Early Domestic (3501-5500 years) | Kazakhstan |
| 183 | ERR3225666 | nc | nc | nc | . | Early Domestic (3501-5500 years) | Kazakhstan |
| 184 | ERR2112780 | . | nc | nc | nc | Early Domestic (3501-5500 years) | Kazakhstan |
| 185 | ERR2112716 | nc | nc | nc | . | Early Domestic (3501-5500 years) | Kazakhstan |
| 186 | ERR2112719 | AG | nc | nc | . | Early Domestic (3501-5500 years) | Kazakhstan |
| 187 | ERR3225669 | AG | nc | nc | . | Early Domestic (3501-5500 years) | Kazakhstan |
| 188 | ERR2112774 | . | . | . | nc | Early Domestic (3501-5500 years) | Kazakhstan |
| 189 | ERR2112772 | AG | . | . | . | Early Domestic (3501-5500 years) | Kazakhstan |
| 190 | ERR2112776 | . | nc | nc | nc | Early Domestic (3501-5500 years) | Kazakhstan |
| 191 | ERR2112778 | nc | GA | nc | nc | Early Domestic (3501-5500 years) | Kazakhstan |
| 192 | ERR2112777 | . | nc | nc | nc | Early Domestic (3501-5500 years) | Kazakhstan |
| 193 | ERR2112771 | . | nc | . | nc | Early Domestic (3501-5500 years) | Kazakhstan |
| 194 | ERR2112773 | AG | . | . | . | Early Domestic (3501-5500 years) | Kazakhstan |
| 195 | ERR2112769 | GG | . | . | . | Early Domestic (3501-5500 years) | Kazakhstan |
| 196 | ERR2112770 | GG | . | . | . | Early Domestic (3501-5500 years) | Kazakhstan |
| 197 | ERR3225563 | . | . | . | . | Ancient (~24000 years) | Russia |
| 198 | ERR982705 | . | . | nc | . | Przewalski | |
| 199 | ERR982714 | AG | . | nc | . | Przewalski | |
| 200 | ERR982717 | AG | . | . | . | Przewalski | |
| 201 | ERR982706 | AG | . | . | . | Przewalski | |
| 202 | ERR982708 | GG | . | . | . | Przewalski | |
| 203 | ERR982716 | . | . | . | . | Przewalski | |

| | A | B | C | D | E | F | G |
|---|---|---|---|---|---|---|---|
| 204 | ERR982718 | . | . | . | . | Przewalski | |
| 205 | ERR982709 | . | . | . | . | Przewalski | |
| 206 | ERR982712 | . | . | . | . | Przewalski | |
| 207 | ERR982715 | GG | . | . | . | Przewalski | |
| 208 | ERR982721 | nc | nc | . | nc | Przewalski | |
| 209 | ERR982713 | . | . | . | . | Przewalski x Domesticated F1 hybrid | |
