# Supplementary material for: Commercial genetic testing for type 2 polysaccharide storage myopathy and myofibrillar myopathy does not correspond to a histopathological diagnosis
Source: Equine Vet J. Author manuscript; Available in PMC 2021 Jul 1. (PMC7937766; doi:10.1111/evj.13345)

Equine Veterinary Journal BEVA

Figure S1: A. The percentage of horses homozygous for the reference allele (blue), heterozygous (yellow) or homozygous (red) for P2, P3a/P3b, or P4 in Warmblood horse. Two subgroups of control WB are presented, both of which had no evident muscle histopathology. The first subgroup consisted of control horses with no potential overlapping clinical signs of PSSM2/MFM. This included horses with no clinical signs (N=11) combined with control horses with signs of shivers/stringhalt (N=16; total N=27). Comparison was made to a second subset of the control group consisting of WB with clinical signs that overlap those of PSSM2/MFM (N=27) [weakness, lameness, atrophy/fasciculations, poor performance, muscle soreness, exertional rhabdomyolysis]. There was no significant difference in the frequency of any of the P variants between the two subgroups of control horses or between horses with no overlapping signs of PSSM2/MFM and PSSM2/MFM horses. **B.** Two subgroups of control WB are presented both of which had no evident muscle histopathology. The first subgroup was < 7 years of age (N=20) and the second ≥ 7 years of age (N=34). There was no significant difference in the frequency of any of the P variants between the two subgroups of control horses or between control horses ≥ 7years of age and PSSM2/MFM horses.

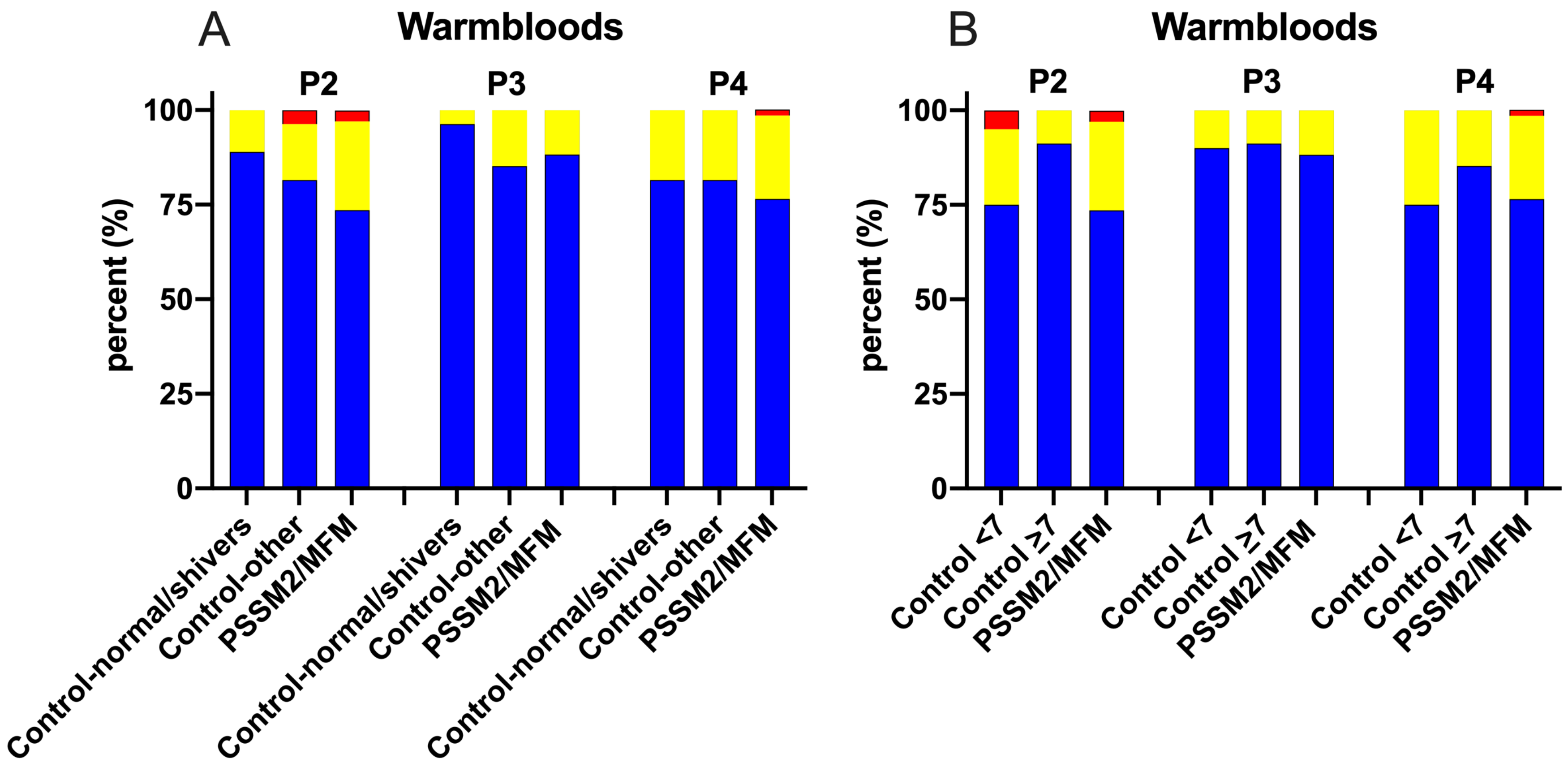

Supplement: Supp Fig 1 [file NIHMS1661435-supplement-Supp_Fig_1.pdf]
