# Supplementary material for: Commercial genetic testing for type 2 polysaccharide storage myopathy and myofibrillar myopathy does not correspond to a histopathological diagnosis
Source: Equine Vet J. Author manuscript; Available in PMC 2021 Jul 1. (PMC7937766; doi:10.1111/evj.13345)
Supplement: Supp Table 3 [file NIHMS1661435-supplement-Supp_Table_3.pdf]

Equine Veterinary Journal BEVA

Table S3: The age, sex, glycogen synthase 1 (*GYS1*) mutation status, number of horses with PAS positive aggregates and number of horses with 4 or more fibres with desmin aggregates for control, type 2 polysaccharide storage myopathy (PSSM2) and myofibrillar myopathy (MFM) presented by breed (Warmblood (WB) and Arabian (AR)).

	**Warmblood**			**Arabian**		
	**Control**	**PSSM2**	**MFM**	**Control**	**PSSM2**	**MFM**
N	54	55	37	30	18	30
Age (y)	8.6 ± 4.3	9.7 ± 4.7	10.1 ± 4.8	12.4 ± 5.9	13.2 ± 3.8	13.9 ± 5.7
Sex	20 f, 33 mc, 1 s	19 f, 34 mc, 2 s	15 f, 19 mc, 2 s, 1 un	22 f, 8 mc	10 f, 8 mc	18 f, 12 mc
GYS1 mutation	neg	neg	neg	nd	9 neg, 9 nd	13 neg, 17 nd
PAS aggregates	0	55	24	0	18	18
Desmin aggregates	0	24	37	0	18	30

f = female; mc = male castrate; s = stallion; un = unknown
neg = negative; nd = not done
GYS1 = glycogen synthase 1
PAS= periodic acid Schiff's
PSSM2 = Polysaccharide storage myopathy type 2
MFM = Myofibrillar Myopathy
