# Supplementary material for: Commercial genetic testing for type 2 polysaccharide storage myopathy and myofibrillar myopathy does not correspond to a histopathological diagnosis
Source: Equine Vet J. Author manuscript; Available in PMC 2021 Jul 1. (PMC7937766; doi:10.1111/evj.13345)
Supplement: Supp Table 4 [file NIHMS1661435-supplement-Supp_Table_4.pdf]

Equine Veterinary Journal BEVA

Table S4: The number of horses of each phenotype, number of horses homozygous for the reference allele, heterozygous or homozygous for each P variant, the percentage of horses possessing the P variant and the variant allele frequencies in control and PSSM2 and MFM Warmbloods (WB) and Arabians (AR) phenotyped on the basis of muscle histopathology. *P*-values are given for comparisons of the prevalence of the variant in PSSM2 or MFM versus controls using genotypic or dominant models. The predicted amino acid substitutions were based upon the variant positions and Ensembl transcript ENSECAT00000076596.1. There were no significant differences in the prevalence of the variants or allele frequencies between control and PSSM2 or MFM horses of either breed.

	N	Homozygous Reference	Heterozygous	Homozygous Alternative	% Horses with 1 or 2 Copies Alternative Allele	Variant Allele Frequency	P-value	
							Genotypic	Dominant
**P2** *MYOT*- rs1138656462								
**Control-WB**	54	46	7	1	15%	0.08		
**PSSM2-WB**	55	41	12	2	25%	0.15	0.23	0.44
**MFM-WB**	37	27	9	1	27%	0.15	0.19	0.82
**Control-AR**	30	24	5	1	20%	0.12		
**PSSM2-AR**	18	14	4	0	22%	0.11	1.00	0.34
**MFM-AR**	30	20	10	0	33%	0.17	0.38	0.23
**P3a/P3b** *FLNC*- rs1139799323 / *FLNC* - rs1142918816								
**Control-WB**	54	49	5	0	9%	0.05		
**PSSM2-WB**	55	49	6	0	11%	0.05	0.74	1.00
**MFM-WB**	37	32	5	0	14%	0.07	0.74	1.00
**Control-AR**	30	29	1	0	3%	0.02		
**PSSM2-AR**	18	17	1	0	6%	0.03	1.00	0.73
**MFM-AR**	30	29	1	0	3%	0.02	1.00	1.00
**P4** *MYOZ3* - rs1142544043								
**Control-WB**	54	44	10	0	19%	0.09		

**PSSM2-WB**	55	43	11	1	22%	0.12	0.81	0.82
**MFM-WB**	37	27	10	0	27%	0.14	0.44	1.00
**Control-AR**	30	26	4	0	13%	0.07		
**PSSM2-AR**	18	15	2	1	17%	0.11	1.00	0.44
**MFM-AR**	30	24	5	1	20%	0.12	0.73	0.73
