# Supplementary material for: Commercial genetic testing for type 2 polysaccharide storage myopathy and myofibrillar myopathy does not correspond to a histopathological diagnosis
Source: Equine Vet J. Author manuscript; Available in PMC 2021 Jul 1. (PMC7937766; doi:10.1111/evj.13345)
Supplement: Supp Table 5 [file NIHMS1661435-supplement-Supp_Table_5.pdf]

Table S5: A. The number of horses with no P variant alleles, one P variant allele, or > 1 P variant allele, and the percentage of horses that possessed > 1 P variant allele across all three loci. P-values are given for comparisons of the number of horses with none, one, or > 1 P variant allele between control and PSSM2 or MFM horses by breed. **B.** The number of horses with no P variant loci, one P variant locus, or > 1 P variant locus, and the percentage of horses that possessed > 1 P variant locus. P-values represent the comparisons of the number of horses with none, one, or > 1 variant loci between control and PSSM2 or MFM horses by breed.

A.		**N**	**Zero Variant Alleles**	**One Variant Allele**	**>1 Variant Allele**	**P-value**
	Control-WB	54	33	18	3	
	PSSM2-WB	55	29	18	8	0.33
	MFM -WB	37	15	18	4	0.15
	Control-AR	30	20	9	1	
	PSSM2-AR	18	10	7	1	0.68
	MFM -AR	30	15	12	3	0.32
B.			**Zero Variant Loci**	**One Variant Locus**	**>1 Variant Locus**	**P-value**
	Control-WB	54	33	19	2	
	PSSM2-WB	55	29	20	6	0.36
	MFM -WB	37	15	19	3	0.13
	Control-AR	30	20	9	1	
	PSSM2-AR	18	10	8	0	0.61
	MFM -AR	30	15	13	2	0.41
