# Supplementary material for: Commercial genetic testing for type 2 polysaccharide storage myopathy and myofibrillar myopathy does not correspond to a histopathological diagnosis
Source: Equine Vet J. Author manuscript; Available in PMC 2021 Jul 1. (PMC7937766; doi:10.1111/evj.13345)
Supplement: Supp Table 6 [file NIHMS1661435-supplement-Supp_Table_6.pdf]

Equine Veterinary Journal BEVA

Table S6: The odds ratio (OR), sensitivity, specificity, positive predictive value (PPV), negative predictive value (NPV) with confidence intervals (CI) for the P2, P3 and P4 variants in PSSM2 or MFM horses by breed. P3a and P3b are reported together as they were in linkage disequilibrium. None of the calculated values reached statistical significance (p<0.05).

	Variant	OR		Sensitivity		Specificity		PPV		NPV	
			CI		CI		CI		CI		CI
PSSM2-WB	P2	1.96	0.78 - 5.18	0.26	0.16 - 0.38	0.85	0.73 - 0.92	0.64	0.43 - 0.80	0.53	0.43 - 0.63
	P3a/P3b	1.20	0.32 - 3.8	0.11	0.05 - 0.23	0.91	0.80 - 0.97	0.55	0.28 - 0.80	0.50	0.40 - 0.61
	P4	1.23	0.49 - 3.31	0.22	0.13 - 0.34	0.81	0.69 - 0.90	0.55	0.35 - 0.73	0.51	0.40 - 0.61
MFM-WB	P2	2.13	0.79 - 6.36	0.27	0.15 - 0.43	0.85	0.73 - 0.92	0.56	0.34 - 0.75	0.63	0.52 - 0.73
	P3a/P3b	1.53	0.45 - 5.25	0.14	0.06 - 0.29	0.91	0.80 - 0.97	0.50	0.24 - 0.77	0.61	0.50 - 0.71
	P4	1.63	0.64 - 4.15	0.27	0.15 - 0.43	0.82	0.69 - 0.90	0.50	0.30 - 0.70	0.62	0.50 - 0.72
PSSM2-AR	P2	1.14	0.21 - 4.16	0.22	0.09 - 0.45	0.80	0.63 - 0.91	0.40	0.17 - 0.69	0.63	0.47 - 0.77
	P3a/P3b	1.71	0.08 - 33.38	0.06	0.001 - 0.13	0.97	0.83 - 1.10	0.50	0.03 - 0.98	0.63	0.49 - 0.76
	P4	1.30	0.29 - 5.36	0.17	0.06 - 0.39	0.87	0.70 - 0.95	0.43	0.16 - 0.75	0.63	0.48 - 0.76
MFM-AR	P2	2.00	0.60 - 7.05	0.33	0.19 - 0.51	0.80	0.63 - 0.91	0.62	0.39 - 0.82	0.55	0.40 - 0.68
	P3a/P3b	2.00	0.05 - 19.60	0.03	0.002 - 0.18	1.97	0.83 - 1.01	1.50	0.03 - 0.98	1.50	0.38 - 0.64
	P4	1.63	0.45 - 5.56	0.20	0.095 - 0.37	0.87	0.70 - 0.95	0.60	0.31 - 0.83	0.52	0.39 - 0.65
