# Supplementary material for: Commercial genetic testing for type 2 polysaccharide storage myopathy and myofibrillar myopathy does not correspond to a histopathological diagnosis
Source: Equine Vet J. Author manuscript; Available in PMC 2021 Jul 1. (PMC7937766; doi:10.1111/evj.13345)
Supplement: Infographic [file NIHMS1661435-supplement-Infographic.pdf]

Do commercial genetic tests for PSSM2 and myofibrillar myopathy (MFM) correspond to a histopathological diagnosis?

Controls

30 Arabians, 54 Warmbloods with normal muscle biopsies

Genotyping for P2, P3, P4 variants

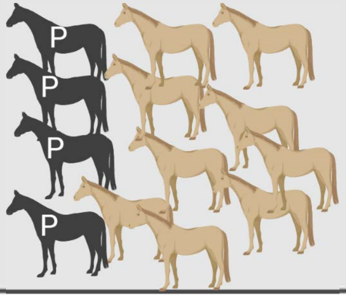

- 30% Arabians, 33% Warmbloods had one of the P variants
- 3% Arabians, 6% Warmbloods had > 1 P variant

No significant differences
Fishers Exact Test P<0.05

MFM/PSSM2

30 Arabians, 68 Warmbloods with PSSM2/MFM by muscle biopsy

Genotyping for P2, P3, P4 variants

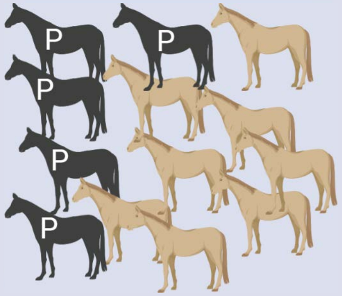

- 40% Arabians, 38% Warmbloods had one of the P variants
- 10% Arabians, 13% Warmbloods had > 1 P variant

Test sensitivity for each P variant was < 33%

P2 and P3 were present in 25 early domestic and modern breeds (public repositories)

A single or combination of commercial genetic test P variants were not statistically associated with a histological diagnosis of PSSM2 and MFM, the means by which these diseases were discovered

Commercial genetic testing for PSSM2 and myofibrillar myopathy do not correspond to a histopathological diagnosis, 2020;00:1-11. https://doi.org.10.1111/evj.13345
